# Diseases of the Intestines

**Published:** 1905-07-29

**Authors:** 


### Progress in Medicine and Surgery.

### DISEASES OF THE INTESTINES.

**Visceroptosis.** — This condition, according to McCallum,[1] is not accompanied by any symptoms as long as the patient is in good health; in neurasthenic conditions the symptoms which occur cannot be alleviated by local measures, a cure for the neurasthenia alone can remove them. Occasionally single local symptoms and secondary complications occur, such as Dietl's crises, hydronephrosis, dilatation of the stomach, constipation or bladder and uterine irritation. On the whole, however, Weir-Mitchell treatment for the neurasthenia is the main indication in visceroptosis. Over 90 per cent. of female neurasthenics suffer from visceroptosis, the main causes of which are, bad standing posture, badly fitting garments, absence of fat, imperfect use of the lower segment of the thorax, and poor tone of the abdominal muscles. The symptoms are abdominal pain in very varied regions, possibly simulating appendicitis, or radiating down the leg, or felt over the liver, malnutrition, anæmia, and occasionally pre-senility and arterio-sclerosis. The shape of the thorax is important and suggestive, especially in the cases of gastroptosis. Dawson [2] describes it as elongated downwards so that there is only a small space between the lower ribs and the iliac crests. The lower ribs are oblique and rigid, and expand but little on inspiration, and the descent of the diaphragm is well marked. Dawson thinks that the tenth ribs are only occasionally "floating," but McCallum says they are found so in a considerable number of cases. The profile view of the anterior abdominal walls shows a double inclined plane, the apex of which McCallum notes is well below the umbilicus, and Dawson describes the hollowing out of the epigastrium. Both writers note the feeble tone of the abdominal muscles and the possibility of making out the curves of a prolapsed stomach, which is aided by inflation or transillumination, or by the use of the fluorescent screen after coating the interior of the stomach with bismuth. McCallum points out also the importance of careful palpation of the solid organs. Dawson, in considering gastroptosis, points out that tight-lacing is a potent cause especially among long-waisted women, and considers that the symptoms usually come on owing to some condition of general ill-health, or as a result of over-distension of the stomach with food, which adds dilatation and motor insufficiency to the pre-existing ptosis. The treatment advised by both writers is essentially identical. Rest in bed from four to eight weeks, cold baths and massage, and careful feeding up are the main points, with exercises to increase the tone of the abdominal muscles and to expand the lower segment of the thorax. McCallum thinks 10 weeks the shortest period for building up the abdominal wall by massage; he notes that the recumbent posture is necessary for feeding these patients, as most of them have some gastric prolapse, so that food cannot pass easily out of the stomach in the erect position of the body. In cases which are improved, rest in the middle of the day or after each meal is necessary, according to Dawson, the foot of the bed being raised 9 to 12 inches. A good exercise, this writer says, consists in lying on the back and then raising the trunk to a sitting posture without raising the heels or using the arms. McCallum notes that in some cases where the kidneys are prolapsed, albumin and casts appear in the urine, which are not due to Bright's disease, but to twisting of the vessels. With regard to surgical measures, Dawson considers gastro-enterostomy useless, and McCallum says that the vast majority of operations for displacement and prolapse of the pelvic organs are unscientific and unnecessary, and those for ptosis of the kidneys and stomach are even more so.

**Ankylostomiasis.**—An examination of the conditions found in the coalfields of Westphalia and Hungary, and the metalliferous mines in Cornwall by Oliver,[3] demonstrate the economic importance of prophylaxis against the parasite. Ankylostomiasis has cost the Miners' Union in Westphalia nearly £200,000. It is not clear how the disease was imported, but its enormous increase in 1900 was due to the watering of the mines, which then became compulsory, owing to the number of explosions which occurred. Todd [4] notes that the necessary conditions for the development of the ova are a moist, pasty medium, a high temperature (*optimum* 78°-86° F.) and the absence of sunlight. Under these conditions the larvæ when hatched become encysted. The hatching of the ova occupies, according to Oliver, 14 days under favourable circumstances, and he and Todd emphasise the fact that as oxygen is necessary for the development of the ova, the complete cycle cannot take place entirely within the body, and the encysted larva is the only form in which the parasite can enter and develop in the intestine. Todd gives two years as the period of life for an adult worm, and says that in temperate climates only men actually working underground are infected. It is not clear that dogs or horses are ever affected, though in monkeys the disease has probably been experimentally induced. As the parasite is found in the mud and sludge of mines, the main indications for prophylaxis are: (1) Thorough ventilation to reduce the temperature to a point below that in which the ovum can develop. Oliver found in Westphalia that by this means alone some mines had been practically freed from the parasite. (2) The provision of galvanised iron buckets for the miners' fæces, which are to be cleaned out above ground every day; (3) Provision of bathrooms, etc., for miners when they leave the mine; (4) Care in feeding, &c., underground. Todd says that it is difficult to get the men to wash before feeding, and that as an alternative the food may be wrapt in paper and held in this while eaten, so that the hands do not come in contact with the mouth. (5) Todd also states that owing to the great resistance of the larvæ when encysted, no practical result has followed attempts to disinfect the mucus with carbolic acid, slaked lime, etc. Oliver found in Westphalia that drinking water acidulated with citric acid was given to the men, but Todd says that this has been proved useless, as the encysted larvæ are unaffected by it. (6) The fæces of all fresh workmen, especially when coming from an infected district, must be examined for ova. Owing to numerous cases of fraud, it is necessary for the men to deposit their fæces in hospital and under efficient supervision. Oliver says that in Westphalia Italian miners are accepted after this test, but Hungarians are not taken on under any circumstances. The diagnosis, according to all observers, rests on the finding of the ova in the fæces. The adult worm is never found, according to Todd, except after a vermifuge. Boycott[5] considers that estimations of hæmoglobin in the blood are of great value, and also states that as regards eosinophilia, anything below 5 per cent. may be taken as normal, 5 to 8 per cent. as doubtful, and anything above 8 per cent. as extremely suspicious. Eosinophilia is most marked in young persons before other symptoms (pallor, lassitude, etc.) have developed—in an accidental infection it was first observed 20 days after contact with the encapsuled larvæ. Bruno, Liefmann, and Mäckel[6] do not altogether agree with Boycott's views. They found in 500 cases of ankylostomiasis most had an eosinophilia of over 8 per cent., and 92 per cent. had one of over 5 per cent. They consider, however, that though with negative stools eosinophilia is of value as showing a probable infection, it is not in itself enough to justify anthelmintic treatment. Moreover, they consider examinations of the fæces easier and more rapid than blood-counts, and have never found any objection to them made by the miners. Leonard,[7] in West Indian negroes found a peculiar pigmentation of the tongue—faint blue to blue-black fine dots on the sides and dorsum—which he considers diagnostic in these patients, though absent in white men. He thinks it is produced by local infection, and is analogous to the mottled pigmentation of the skin occurring in "ground itch," where the larvæ gain access to the host through his skin. All observers note the frequent absence of anæmia in those harbouring the worm; Oliver found in Hungary that a kind of tolerance was established after a time; though many men were still unfit for work, the disease tended to become milder in type. The treatment in Cornwall is mainly thymol, in Germany filix mas is given, though Oliver notes that neuroretinitis, with blindness, has been known to follow its exhibition in large doses. In Hungary, Oliver found experiments in progress with an infusion of aceric anthelmintica, which is much used in Abyssinia. Todd gives a formula composed of turpine, sulphur, and condurango, which has recently been tried at Seraing. He says the slight cases are often the most obstinate, repeated doses being required to expel all the worms.

1 Brit. Med. Jour., Feb. 18, 1905, p. 345. 2 Clin. Jour., Jan. 4, 1905, p. 183. 3 Lancet, Dec. 10, 1904, p. 1635. 4 Glasg. Med. Jour., Nov. 1904, p. 365. 5 Med. Chron., Feb. 1905, p. 309. 6 Med. Rec., March 4, 1905, p. 249. 7 Ibid., p. 338.

*(To be continued.)*

